# Identification of a major quantitative trait locus underlying salt tolerance in ‘Jidou 12’ soybean cultivar

**DOI:** 10.1186/s13104-018-3202-3

**Published:** 2018-02-05

**Authors:** XiaoLei Shi, Long Yan, ChunYan Yang, WeiWen Yan, David Octor Moseley, Tao Wang, BingQiang Liu, Rui Di, PengYin Chen, MengChen Zhang

**Affiliations:** 1 Institute of Cereal and Oil Crops, Hebei Academy of Agricultural and Forestry Sciences/Shijiazhuang Branch Center of National Center for Soybean Improvement/Huang-Huai-Hai Key Laboratory of Biology and Genetic Improvement of Soybean, Ministry of Agriculture/Key Laboratory of Crop Genetics and Breeding, Shijiazhuang, 050035 China; 2 Department of Crop, Soil and Environment Sciences, University of Arkansas, Fayetteville, AR 72701 USA

**Keywords:** Soybean, Salt-tolerant, QTL, STR, SPAD

## Abstract

**Background:**

Identification of the quantitative trait locus (QTL) underlying salt tolerance is a prerequisite for marker-assisted selection in the salt-tolerant breeding process.

**Methods:**

In this study, the recombinant inbred lines derived from the salt-tolerant elite soybean cultivar ‘Jidou 12’ and the salt-sensitive elite cultivar ‘Ji NF 58’ were used to identify the QTL associated with salt tolerance, using both salt tolerance rating (STR) and leaf chlorophyll content (SPAD) as indicators.

**Results:**

A major salt-tolerant QTL, which was flanked by SSR markers GMABAB and Barcsoyssr_03_1421 on chromosome 3, was identified based on single-marker regression, single trait composite interval mapping, and multiple interval mapping analysis. For STR, the LOD ranged from 19.8 to 20.1; R^2^ ranged from 44.3 to 44.7%; and the additive effect ranged from 0.876 to 0.885 among the three mapping methods. For SPAD, the LOD ranged from 10.6 to 11.0; R^2^ ranged from 27.0 to 27.6%; and the additive effect ranged from 1.634 to 1.679 among the three mapping methods.

**Conclusions:**

In this study, a major QTL conditioning salt tolerance on chromosome 3 was identified. The DNA markers closely associated with the QTLs might be useful in marker-assisted selection for soybean salt tolerance improvement in Huanghuaihai, China.

**Electronic supplementary material:**

The online version of this article (10.1186/s13104-018-3202-3) contains supplementary material, which is available to authorized users.

## Background

Salinity is one of the major abiotic stress factors that significantly reduces crop yields. Approximately 20% of irrigated land is affected by salt stress around the world [1]. It has been estimated that 6.62% of the arable land was affected by salt stress in China. Development of salt-tolerant crops will be required to improve the sustainability of crop production.

Soybeans are an important crop for food, feed, and biodiesel production due to their high oil and protein content. Soybean is considered a moderately salt-sensitive crop [2]. Salt stress will damage all developmental stages of soybean, including germination, plant growth [3], nodule formation [4], and seed yield [5]. The tolerant soybean cultivars had 37% higher yields than the susceptible cultivars [6]. Hence, development of salt tolerant soybean cultivars will be an effective way to maintain sustainable production in salt stressed regions. However, because there was a lack of a simple and cost effective way to precisely evaluate a large number of lines for salt tolerance during the selection process in breeding, the development of salt-tolerant soybean cultivars was hampered for a long time. Therefore, marker-assisted selection (MAS) may be a particularly good method for the salt tolerance breeding process. A prerequisite for MAS is knowledge of the genomic location and genetic effect of the major quantitative trait locus (QTL), which conditions salt tolerance in soybean. Two QTLs associated with salt tolerance have been mapped on chromosome 3 (Linkage group N) and chromosome 18 (Linkage group G). It has been reported that wild soybean accession PI 483463 [7], wild soybean accession JWS156-1 [8], wild soybean accession W05 [9], soybean cultivar S-100 [10], soybean cultivars FT-Abyara and Jin dou No. 6 [11], and soybean cultivar Tiefeng 8 [12, 13] contain the salt tolerant allele on chromosome 3. Additionally, soybean cultivar Kefeng No.1 [14] contains the tolerant allele on chromosome 18.

The Huanghuaihai region was the second most important area for soybean production in China. The area of saline land is approximately 4.67 million hectares in the Huanghuaihai region [15]. Identification of the QTL conditioning salt tolerance in the elite cultivars that are adapted to this region will aid soybean breeding in this special area. Jidou 12 was an elite soybean cultivar in the Huanghuaihai region [16], widely used as a breeding parent [17] for its high yield [18], high protein content [19], and soybean mosaic virus (SMV) tolerance [20]. ‘Ji NF 58’ was a soybean cultivar in the Huanghuaihai region containing high oil content [16]. Based on the Na^+^ content analysis in different organs at the seeding stage, ‘Jidou 12’ was considered to be a salt-tolerant cultivar while ‘Ji NF 58’ was a salt-susceptible soybean cultivar [15].

In this study, QTL analyses for salt tolerance were performed in a RIL population derived from ‘Jidou 12’ × ‘Ji NF 58’, to identify the QTL conditioning salt tolerance in ‘Jidou 12’ and to facilitate MAS soybean salt tolerance breeding in Huanghuaihai, China.

## Methods

### Plant materials

A RIL population, consisting of 156 lines derived from the cross ‘Jidou 12’ × ‘Ji NF 58’, was used to identify the QTL associated with salt tolerance. ‘Jidou 12’ was an elite high protein content cultivar, while ‘Ji NF 58’ was an elite high oil content cultivar. They were both bred at the Institute of Cereal and Oil crops, Hebei Academy of Agricultural and Forestry Sciences, Shijiazhuang, China. Seeds for the F_2_ generation were collected from a single F_1_ plant in late September 2002. The RIL were advanced to the F_9:10_ generations by single-seed descent without any particular selection method.

### Evaluation of salt tolerance

**Fig. 1:**
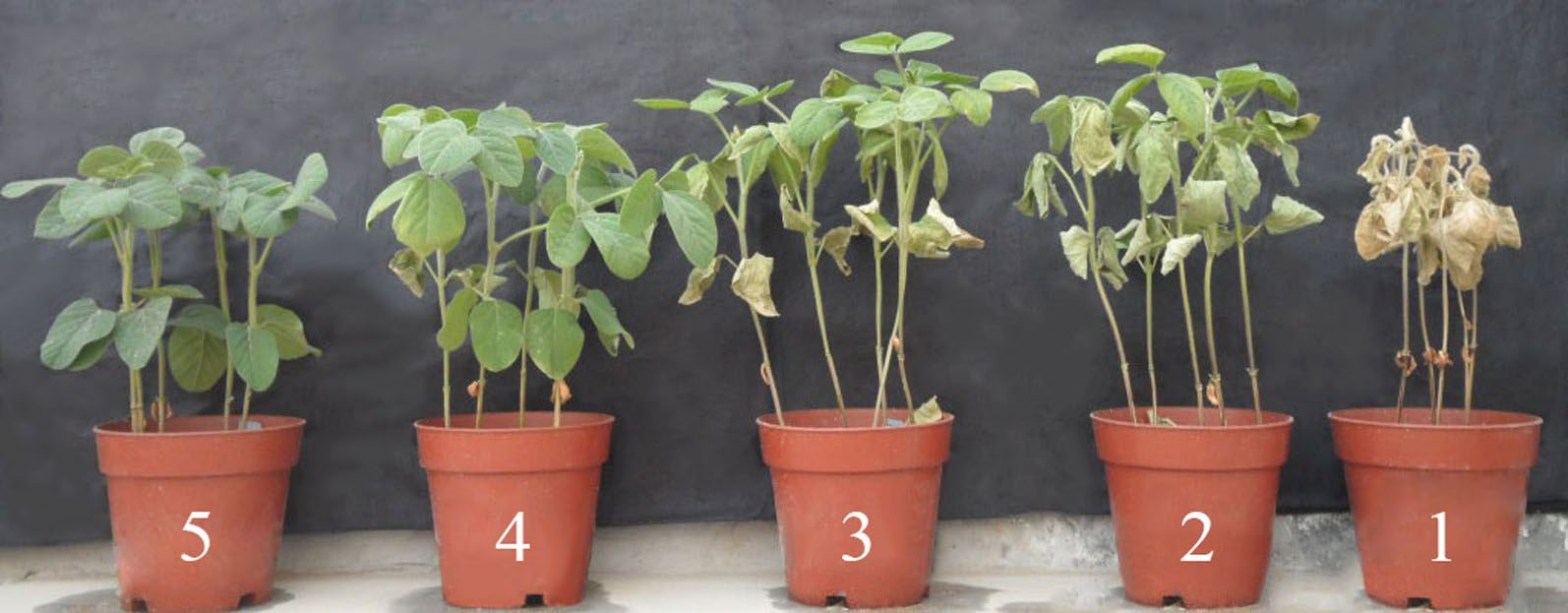
Salt tolerance rating (STR) of the progeny derived from ‘Jidou 12’ × ’Ji NF 58’. 1, plants completely died; 2, two-thirds or more leaves showed chlorotic symptoms or only upper leafs survived; 3, half or fewer leaves showed chlorotic symptoms; 4, one-third or fewer leaves showed chlorotic symptoms; 5, plants grew normally

A salt-water flood method [21] was used to evaluate the salt tolerance of the RIL and their parents. In brief, five healthy plants of each cultivar/line were grown in a pot (15 × 15 cm) filled with sand, and 8 pots were placed in a large plastic container (65 × 40 × 15 cm) containing half-strength Hoagland and Arnon nutrient solution. Two weeks after planting, the soybean plants were initially treated with 60 mM NaCl. After 3 days, NaCl concentration was raised to 150 mM. The NaCl solution was replaced every 3 days and the concentration was maintained at the same level until the end of the experiment. The natural sunlight in the greenhouse was supplemented by high-pressure sodium light to provide 14-h photoperiod. The temperature was controlled by air conditioning to maintain the temperature at approximately 25 ± 2 °C. When the salt-sensitive soybean cultivar, Clark, showed clearly salt toxicity symptom (complete death), the salt tolerance rating (STR) for each genotype was evaluated by the chlorotic leaf of soybean plants. The STR scale was divided into five grades, ranging from 1 to 5 [11] (Fig. 1). Meanwhile, the chlorophyll content (SPAD value) of leaf from the same node number was measured using a chlorophyll meter (Konica Minolta SPAD-502). This SPAD value is proportional to the chlorophyll content in leaves. The evaluation for the RIL and parents was carried out in a greenhouse at the Institute of Cereal and Oil crops, Hebei Academy of Agricultural and Forestry Sciences, Shijiazhuang, China. The experiment consisted of a random block design with three replications. The entire experiment was carried out two times, independently of each other.

### DNA extraction and SSR analyses

The young leaves from single plants for each F_9_ plant were collected for DNA extraction. Next, 357 SSR markers that were well distributed throughout the 20 soybean genetic linkage map [22–24] were selected to test the polymorphism between the parents of the population. Subsequently, 118 polymorphic SSR markers were used to analyze the population (Additional file 1: Table S1). SSR markers information were obtained from the SoyBase website of the USDA, ARS Genome Database (http://soybase.agron.iastate.edu/). DNA extraction and marker analysis were conducted following the protocols described by Wang et al. [25].

### Data analysis

The statistical analyses for STR and SPAD were conducted using JMP Pro 11 (SAS Institute, Cary, NC). The genetic linkage map based on the polymorphism markers was constructed by using the Map Manager program QTXb20 (http://mapmgr.roswellpark.org/mapmgr.html), with the selection of Kosambi for the Map Function and a 0.01 probability for the linkage criterion. The candidate QTL controlling salt tolerance was identified by single-marker regression (SMR), single trait composite interval mapping (CIM), and single trait multiple interval mapping (MIM) analyses using QGene 4.3.10 [26]. QTL LOD levels higher than 2.5 were significant. The LOD plots were created by MapChat 2.2 [27] based on the data from QTXb20 and QGene.

## Results and discussion

**Fig. 2:**
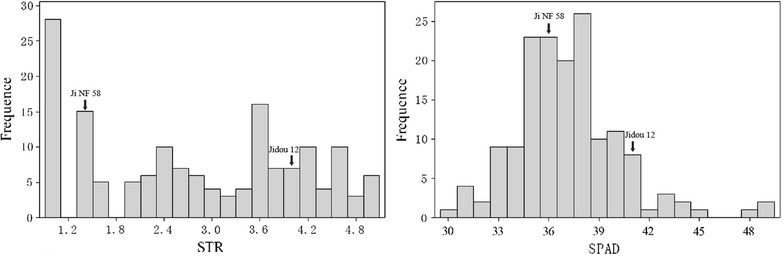
Frequency distribution of salt tolerance rating (STR) and leaf chlorophyll content (SPAD) for RILs derived from ‘Jidou 12’ × ’Ji NF 58’

‘Jidou 12’ showed significantly (P ≤ 0.01) higher salt tolerance than ‘Ji NF 58’ for both STR and SPAD. The STR of ‘Jidou 12’ was 4.0 while it was 1.5 for ‘Ji NF 58’. The SPAD of ‘Jidou 12’ was 40.6 while it was 36.5 for ‘Ji NF 58’. Continuous distribution and transgressive segregation of the STR and SPAD were observed in the RILs (Fig. 2). For the 156 RILs, the STR ranged from 1.0 to 5.0, with an average of 2.79, and the SPAD ranged from 30.5 to 49.2, with an average of 37.1. There was a significant positive correlation (r = 0.659, P ≤ 0.01) between the STR and SPAD values.

**Fig. 3:**
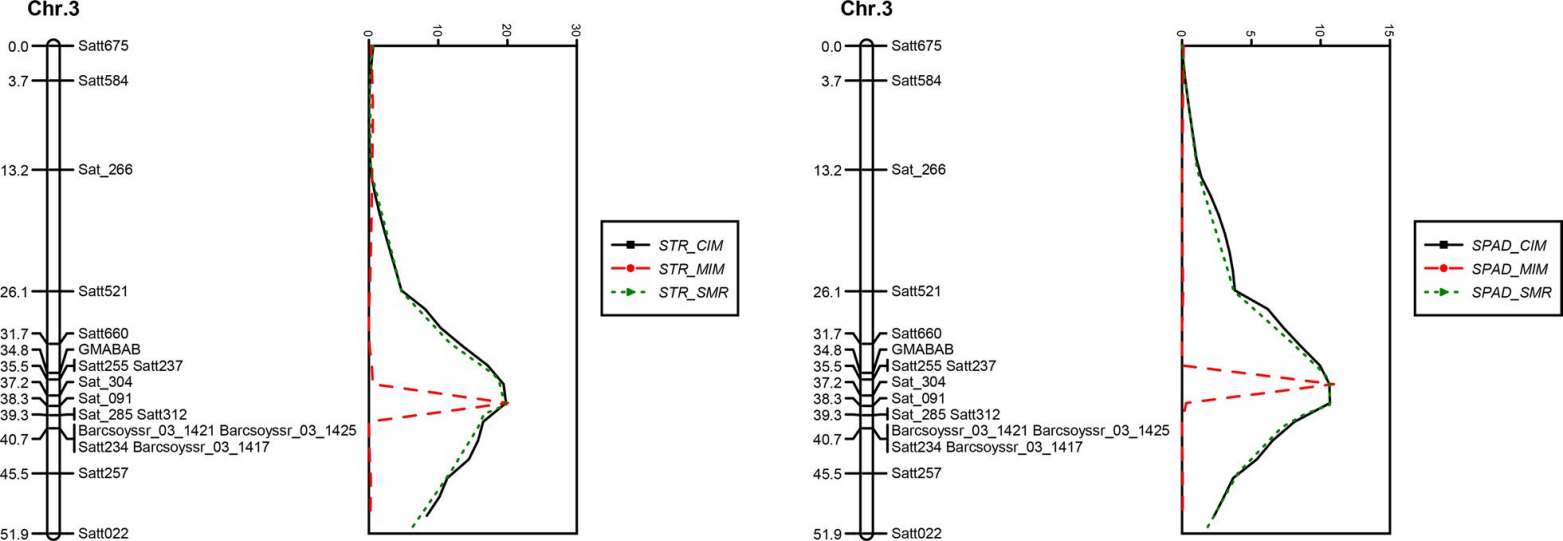
Genetic linkage map of markers on chromosome 3 and the LOD score of QTL associated with salt tolerance rating (STR) and leaf chlorophyll content (SPAD); the QTL analysis of RILs derived from ‘Jidou 12’ × ’Ji NF 58’ was based on single-marker regression (SMR), single trait composite interval mapping (CIM), and multiple interval mapping (MIM) methods

The linkage map of ‘Jidou 12’ × ‘Ji NF 58’, consisting of 118 SSR markers and covering a total distance of 1008.4 cM on 20 linkage groups, was established. The average distance between two markers was 8.5 cM. Only one QTL for both STR and SPAD was detected on chromosome 3, using the averaged data by SMR, CIM, and MIM (Table 1 and Fig. 3). For STR using SMR, this QTL was closely linked with SSR marker Sat_091. The LOD was 19.8, and the QTL could explain 44.3% of the total phenotype variance. The additive effect of this QTL was 0.876. For STR using CIM, this QTL was flanked by SSR markers GMABAB and Barcsoyssr_03_1421 while it was closely linked with SSR marker Sat_091. The LOD was 19.8, and the QTL could explain 44.3% of the total phenotype variance. The additive effect of this QTL was 0.877. For STR using MIM, this QTL was closely linked with SSR marker Sat_091. The LOD was 20.1, and the QTL could explain 44.7% of the total phenotype variance. The additive effect of this QTL was 0.885. For SPAD using SMR, this QTL was closely linked with SSR marker Sat_091. The LOD was 10.7, and the QTL could explain 27.0% of the total phenotypic variance. The additive effect of this QTL was 1.634. For SPAD using CIM, this QTL was flanked by SSR markers GMABAB and Barcsoyssr_03_1421 and was closely linked with SSR marker Sat_091. The LOD was 10.7, and the QTL could explain 27.0% of the total phenotype variance. The additive effect of this QTL was 1.637. For SPAD using MIM, this QTL was closely linked with Satt237 and Satt255. The LOD was 11.0, and the QTL could explain 27.6% of the total variance. The additive effect of this QTL was 1.679.

**Table 1:** QTL analysis of salt tolerance rating (STR) and leaf chlorophyll content (SPAD) values for RIL derived from ‘Jidou 12’ × ’Ji NF 58’ using different mapping method

Trait	Method	Nearest marker	Position (cM)	LOD	R^2^(%)^a^	Additive^b^
STR	SMR	Sat_091	38.3	19.8	44.3	0.876
	CIM	Sat_091	38.3	19.8	44.3	0.877
	MIM	Sat_091	38.3	20.1	44.7	0.885
SPAD	SMR	Sat_091	38.3	10.7	27.0	1.634
	CIM	Sat_091	38.3	10.7	27.0	1.636
	MIM	Satt237/Satt255	35.5	11.0	27.6	1.679

*SMR* single-marker regression, *CIM* composite interval mapping, *MIM* multiple interval mapping

^a^R^2^ was the percentage of the phenotype variance explained by the QTL

^b^The estimated additive effect of the alleles of ‘Jidou 12’

The salt tolerance of the population (‘Jidou 12’ × ‘Ji NF 58’) was tested using STR and SPAD. Based on the phenotype distribution analysis, transgressive segregation of both STR and SPAD were observed in this population. There were 43 and 71 lines that expressed lower salt tolerance than their sensitive parent ‘Ji NF 58’ in terms of STR and SPAD values, respectively. Additionally, there were 33 and 16 lines that expressed higher salt tolerance than ‘Jidou 12’ in terms of STR and SPAD values, respectively. These results implied that there should be more than one gene controlling salt tolerance in this population. However, only one major QTL, explaining 27–44% of phenotypic variance, was identified as a result of the mapping study. In another study, a similar result was observed in the populations of ‘FT-Abyara’ × ‘C01’ and ‘Jin dou No. 6’ × ‘0197’. In both cases, transgressive segregation was observed while only one QTL was identified [11]. It could be inferred that the lack of precise evaluation of salt tolerance was the major reason for these results, for which environmental effects and random error could be responsible. Two reasons for this lack of precision are that salt concentration has a gradient in both horizontal and vertical directions and human error while conducting the experiment [11]. To enhance the effectiveness of salt tolerance breeding, MAS was recommended for use in commercial breeding. There is a strong relationship between marker alleles and salt tolerance [10]; therefore, MAS will help compensate for the reduced precision in evaluating lines for salt tolerance.

In this study, a major QTL conditioning salt tolerance on chromosome 3 was identified using SMR, CIM, and MIM mapping methods. Several linkage and association analysis [7–13] experiments, consisting of different study populations, strongly suggested that this QTL is stable. Meanwhile, this QTL has great application value. Lee et al. [10] examined the association between the salt tolerance and the flanking marker alleles of salt tolerance QTL in US cultivars descending from S-100 through 60 years of breeding by pedigree tracking. As a result, alleles at the Sat_091 and Satt237 loci from S-100 is always associated with salt tolerance in descendants. Following other research of soybean salt tolerance, our study has two contributions for soybean breeding. First, the QTL and its linked markers identified in this study are helpful to MAS in developing salt tolerant soybean cultivars.

This will assist in developing salt tolerant soybean cultivars that produce high yield, high protein content, and high oil content for the Huanghuaihai region in China, to which the parents of this study are well adapted. ‘Jidou 12’ has often been used as a parent in soybean breeding and at least four cultivars were released in China. At least three other sister lines of ‘Ji NF 58’ were released in the last decade. The second contribution of this study is that the salt tolerant parent ‘Jidou 12’ should contain a novel salt tolerant allele on chromosome 3 because there was no common parent found in other studies.

It has been reported that the gene under this QTL should be *GmSALT3*, and *Glyma03g32900* was regarded as the candidate causal gene underlying *GmSALT3*, based on the analysis of the soybean reference genome obtained from Williams 82. Higher expression of the *Glyma03g32900* in the soybean root resulted in lower accumulations of Na^+^, K^+^, and Cl^−^ in the shoot under salt stress and showed significantly higher root fresh weights than the control. Transfer of *Glyma03g32900* through *Agrobacterium*-mediated transformation into a soybean cultivar significantly enhanced its salt tolerance [9, 13, 28, 29]. In this study, the QTL was mapped to a narrowed region on chromosome 3 containing *Glyma03g32900*, based on the sequence of Williams 82 [30]. This result suggested that *Glyma03g32900* should also be the gene controlling salt tolerance in ‘Jidou 12’. To ensure the validity of the candidate gene, the expression difference of *Glyma03g32900* between the parents and the progeny, segregating for salt tolerance and sensitivity, should be examined.

## Conclusion

The QTL for STR and SPAD was consistently identified on the soybean chromosome 3, and identification of the DNA markers closely associated with the QTLs will significantly improve the efficiency in selecting for salt tolerance in soybean.

## Additional file

**Additional file 1: Table S1.** 118 polymorphic SSR markers used in the ‘Jidou 12’ × ‘Ji NF 58’ population.

## Abbreviations

QTL: quantitative trait locus
RIL: recombinant inbred lines
STR: salt tolerance rating
SPAD: leaf chlorophyll content
MAS: marker-assisted selection
SMR: single-marker regression
CIM: composite interval mapping
MIM: multiple interval mapping
