# Supplementary material for: Identification of a major quantitative trait locus underlying salt tolerance in ‘Jidou 12’ soybean cultivar
Source: BMC Res Notes. 2018 Feb 5;11:95. doi: 10.1186/s13104-018-3202-3 (PMC5800283; doi:10.1186/s13104-018-3202-3)
Supplement: Supplementary file 1 — Additional file 1: Table S1. 118 polymorphic SSR markers used in the ‘Jidou 12’ × ‘Ji NF 58’ population. [file 13104_2018_3202_MOESM1_ESM.docx]

Supplementary table 1. 118 polymorphic SSR markers used in the ‘Jidou 12’ × ‘Ji NF 58’ population

| Chromosome | Polymorphic SSR markers | Number |
| --- | --- | --- |
| 1 | Satt267 Sat_345 Satt267 Sat_345 | 4 |
| 2 | Sat_135 Satt141 Satt611 Satt604 Satt005 Satt579 Satt350 Satt041 | 8 |
| 3 | Satt675 Satt584 Sat_266 Satt521 Satt660 GMABAB Satt237 Satt255 Sat_304 Sat_091 Satt312 Sat_285 Satt022 SOYSSR_03_1417 SOYSSR_03_1421 SOYSSR_03_1425 Satt257 Satt022 | 18 |
| 4 | Sat_367 Satt578 Satt294 Satt713 | 4 |
| 5 | Satt684 Sat_368 Satt276 Sat_344 Satt382 Satt526 Satt236 Sat_271 | 8 |
| 6 | Satt281 Satt457 Satt376 Satt286 Satt557 Satt289 Sat_251 Satt460 Satt316 Satt371 | 10 |
| 7 | Satt728 Sat_422 Satt346 Satt308 | 4 |
| 8 | Satt632 Satt424 Satt437 Sat_382 Sat_294 | 5 |
| 9 | Satt055 Satt167 Satt326 | 3 |
| 10 | Sat_196 Satt358 Sat_318 Satt420 Sat_341 Satt477 Satt331 Satt592 Satt581 | 9 |
| 11 | Sat_272 Satt509 Satt197 Satt597 Satt583 | 5 |
| 12 | Satt192 Satt317 Satt434 | 3 |
| 13 | Sat_262 Satt269 Satt252 Satt335 | 4 |
| 14 | Satt168 Satt556 Satt066 Satt063 Satt726 Satt687 | 6 |
| 15 | Satt384 Satt651 Satt491 Satt268 Satt185 | 5 |
| 16 | Satt596 Sat_151 Sat_350 Satt244 | 4 |
| 17 | Sat_222 Satt082 Sat_001 Sat_022 | 4 |
| 18 | Satt352 Satt427 Satt594 Satt612 Sct_199 Sat_372 | 6 |
| 19 | Satt156 Sat_286 Satt229 Satt373 | 4 |
| 20 | Satt496 Satt354 Sat_105 Sat_299 | 4 |
